# Supplementary material for: Profiles of Volatile and Phenolic Compounds as Markers of Ripening Stage in Candonga Strawberries
Source: Foods. 2021 Dec 14;10(12):3102. doi: 10.3390/foods10123102 (PMC8701158; doi:10.3390/foods10123102)
Supplement: Supplementary file 1 [file foods-10-03102-s001.zip › foods-1483945-supplementary.pdf]

foods

MDPI

Supplementary File:

Table S1. Volatile metabolites detected in cv "Sabrosa" strawberries and their identification codes.

Metabolite	Code	RIt/RIsp	ID	Metabolite	Code	RIt/RIsp	ID
**Esters**				**Aldehydes**			
Methyl propionate	E1	872/872	RI/MS/S	Hexanal	Adl1	1084/1084	RI/MS/S
Methyl butyrate	E2	989/989	RI/MS/S	2-Hexenal	Ald2	1242/1242	RI/MS/S
Methyl isovalerate	E3	1021/1022	RI/MS/S	Nonanal	Ald3	1404/1404	RI/MS/S
Ethyl butyrate	E4	1037/1037	RI/MS/S	Benzaldehyde	Ald4	1533/1533	RI/MS/S
Isopropyl butyrate	E5	1042/1042	RI/MS/S	Dodecanal	Ald5	1716/1716	RI/MS/S
Butyl acetate	E6	1074/1075	RI/MS/S	**Alcohols**			
Methyl pentanoate	E7	1087/1087	RI/MS/S	1-Hexanol	Al1	1365/1365	RI/MS/S
Ethyl pentanoate	E8	1121/1122	RI/MS/S	*trans*-3-Hexen-1-ol	Al2	1375/1376	RI/MS/S
Methyl hexanoate	E9	1204/1204	RI/MS/S	*cis*-3-Hexen-1-ol	Al3	1393/1394	RI/MS/S
Butyl butyrate	E10	1235/1232	RI/MS/S	*trans*-2-Hexen-1-ol	Al4	1416/1416	RI/MS/S
Ethyl hexanoate	E11	1249/1249	RI/MS/S	1-Octanol	Al5	1561/1561	RI/MS/S
Isoamyl butyrate	E12	1277/1267	RI/MS/S	**Acids**			
Hexyl acetate	E13	1284/1285	RI/MS/S	Propanoic acid	Ac1	1545/1545	RI/MS/S
Methyl 2-hexenoate	E14	1288/1284	RI/MS	2-Methylpropionic acid	Ac2	1573/1573	RI/MS/S
cis-3-Hexen-1-ol acetate	E15	1326/1326	RI/MS/S	Butyric acid	Ac3	1631/1631	RI/MS/S
trans-2-Hexen-1-ol acetate	E16	1344/1344	RI/MS/S	2-Methylbutanoic acid	Ac4	1676/1676	RI/MS/S
Methyl octanoate	E17	1396/1398	RI/MS/S	Hexanoic acid	Ac5	1848/1848	RI/MS/S
trans-2-Hexen-1-ol propionate	E18	1400/1392	RI/MS	Heptanoic acid	Ac6	1952/1952	RI/MS/S
n-Hexyl isobutyrate	E19	1420/1353	RI/MS/S	Octanoic acid	Ac7	2073/2073	RI/MS/S
trans-2-Hexenyl butyrate	E20	1478/1476	RI/MS/S	Nonanoic acid	Ac8	21762176	RI/MS/S
Methyl 3-(methylthio) propionate	E21	1529/1525	RI/MS/S	Decanoic acid	Ac9	2278/2278	RI/MS/S
Hexyl hexanoate	E22	1609/1608	RI/MS/S	**Terpenes**			
n-Octyl isobutyrate	E23	1548/1547	RI/MS/S	Linalool	T1	1553/1553	RI/MS/S
Octyl 2-methylbutyrate	E24	1649/1635	RI/MS	β-Farnesene	T2	1667/1667	RI/MS
Methyl 3-hydroxyhexanoate	E25	1653/1641	RI/MS/S	α-Terpineol	T3	1702/1690	RI/MS/S
Benzyl acetate	E26	1729/1731	RI/MS/S	β-Damascenone	T4	1834/1835	RI/MS/S
**Furanones**				Nerolidol	T5	2055/2055	RI/MS/S
Mesifurane	F1	1592/1600	RI/MS/S	**Lactones**			
Furaneol	F2	2042/2042	RI/MS/S	γ-Octalactone	L1	1919/1919	RI/MS/S
trans-γ-Jasmolactone	F3	2188/2181	RI/MS	γ-Decalactone	L2	2158/2158	RI/MS/S
**Others**				γ-Dodecalactone	L3	2387/2384	RI/MS/S
Acetophenone	O1	1658/1658	RI/MS/S				

RI_t: Relative retention indices on polar column reported in literature by www.pherobase.com; www. flavornet.org; www.ChemSpider.com; webbook.nist.gov; RI_{sp}: Relative retention indices calculated against n-alkanes (C_8–C_{40}) on HP-Innowax column; ID: Identification method as indicated by the following: RI: Kovats retention index on a HP-Innowax column; MS: NIST and Wiley libraries spectra; S: co-injection with authentic standard compounds on the HP-Innowax column.
